# Down regulation by a low-zinc diet in gene expression of rat prostatic thymidylate synthase and thymidine kinase

**DOI:** 10.1186/1743-7075-5-12

**Published:** 2008-05-18

**Authors:** Yuko Ishikawa, Hideki Kudo, Satoe Suzuki, Nahoko Nemoto, Shuji Sassa, Shinobu Sakamoto

**Affiliations:** 1 Institute of Nutrition Sciences, Kagawa Nutrition University, Sakado-city, Saitama 350-0288, Japan; 2 Institute of Medical Technology, Bunkyogakuin University, Tokyo 113-8668, Japan; 3 Medical Research Institute, Tokyo Medical and Dental University, Tokyo 113-8510, Japan

## Abstract

**Background:**

Zinc has a wide spectrum of biological activities and its deficiency is related to various abnormalities of cell metabolism.

**Methods:**

Wistar male rats, at age of 4 weeks, were fed a low-zinc diet for six weeks. The levels of bromodeoxyuridine incorporated into the prostatic DNA and the mRNA expression levels of prostate thymidylate synthase and thymidine kinase were examined.

**Result:**

The low-zinc diet caused a marked reduction in the body growth and organ weights, resulted in a low hematopoiesis, hypo-albuminemia and hypocholesterolemia. Although there were few differences in plasma biochemical markers, plasma levels of luteinizing hormone and testosterone were reduced by the low-zinc diet. Bromodeoxyuridine-immunoreactive (S-phase) cells and mRNA expression levels of thymidylate synthase and thymidine kinase in the prostate cells were markedly affected by the low-zinc diet.

**Conclusion:**

A low-zinc diet appears to reduce the body growth and organ weights including prostate, causing low plasma levels of luteinizing hormone and testosterone and reduction in prostate DNA replication in growing-rats.

## Background

Zinc has a wide spectrum of biological activities and its deficiency has been related to various abnormalities of cell metabolism. Zinc is present in the cell nucleus, nucleolus and chromosomes, and zinc stabilizes the structure of DNA, RNA and ribosomes [1]. More than 200 enzymes require zinc as a functional component, and these enzymes affect most major metabolic processes.

Thymidylate synthase (TS) and thymidine kinase (TK) is key enzymes involved in *de novo *and salvage pathways of pyrimidine metabolism, respectively. As previously reported [2], a synthetic analogue of ethinyltestosterone (danazol) reduced the weight of prostate with decline of plasma levels of luteinizing hormone and testosterone, resulting in lowering activities of prostate TS and TK, and decreasing the number of bromodeoxyuridine (BrdU)-immunoreactive (S-phase) cells in the rat prostate.

In the present study, we investigated the effects of a low-zinc diet on body growth, organ weights, hemograms and plasma levels of biochemical markers and hormones, and the expression levels of TS and TK mRNA and BrdU-immunoreactive cells in the prostate of growing rats.

## Methods

### Animals and experimental procedures

Male rats of the Wistar strain (CLEA Japan, Tokyo, Japan), 3 weeks of age, were used. Throughout the experiment, all rats were housed under controlled conditions (24 ± 0.5°C and 12 hours of light from 6 A.M. to 6 P.M.). The animals were permitted free access to normal commercial feed (AIN-76A, Oriental Yeast, Tokyo, Japan), which was a purified diet based on casein as a sole source of protein, and tap water *ad libitum *at the animal research center of the Kagawa Nutrition University (Saitama, Japan). All experimental procedures conformed to the regulations described in the Guide to the Care and Use of Laboratory Animals of the U.S. National Institutes of Health (NIH).

At 4 weeks of age, the animals were divided into two groups of 10 rats each, i.e. a control group and an experimental group. The animals in the control and experimental groups were each fed 10 g per day of the control diet (AIN-76A) and the low-zinc diet (F2ZnDD, Oriental Yeast) [3], respectively, and weighed every 7 days. Five animals out of each group were given a single intravenous injection of bromodeoxyuridine (BrdU; 10 ml/kg body weight, Cell proliferation kit, Amersham, RPN 20, Lot 315827A, Pack 318198, UK) into the tail vein, 2 hours before autopsy. Each rat was bled, at the age of 10 weeks, by cardiac puncture under deep anesthesia (1.5 g urethane/kg body weight, Merck, Darmstadt, Germany), and the spleen, liver, kidney, adrenals, testis, ventral prostate and femur were removed and weighed. Each prostate obtained was immediately fixed in a 10% formaldehyde buffer solution (pH 7.2) to evaluate the S-phase cells in the prostate. Examinations of hemograms, plasma levels of hormones, and biochemical markers were performed later (SRL, Inc., Tokyo, Japan).

### Immunohistochemistry with BrdU in prostate

The levels of BrdU incorporated into the cellular DNA was determined by a monoclonal anti-BrdU antibody; the measurement proceeded according to the manufacture's instructions. Six sections of each prostate were randomly chosen and BrdU-immunoreactive cells were counted among 400 cells per section. The results are expressed in term of BrdU-immunoreactive (S-phase) cells as a percentage of total cells.

### Prostatic thymidylate synthase and thymidine kinase mRNA

Thymidylate synthase (TS; EC 2.1.1.45) and thymidine kinase (TK; EC 2.7.1.21) catalyze the formation of deoxythymidine monophosphate (dTMP) by the methylation of deoxyuridine monophosphate (dUMP) with the concomitant conversion of *N*^5^,*N*^10^-methylenetetrahydrofolic acid to 7,8-dihydrofolic acid *via *the *de novo *pathway and the phosphorylation of thymidine *via *the salvage pathway, respectively [4]. RT-PCR was performed for quantitative analysis of TS and TK mRNA levels in the prostate. Total RNA was extracted from each prostate sample with a QuickPrep™ Total RNA Extraction Kit (Amersham Pharmacia Biotech, Buckinghamshire, UK). Reverse transcription was performed using oligo (dT) primers [0.5 ml oligo (dT)_12–18 _primers (1.0 μg/ml) (GIBCO BRL, Gaithersburg, MD, USA)] with a SUPERSCRIPT™ Preamplification System (GIBCO BRL) according to the supplier's instructions. Once the cDNA copy had been created using the mRNA template, the PCR was conducted immediately, as outlined below. Alternatively, the cDNA was stored at -20°C until use. The PCR was performed with recombinant Taq DNA polymerase (Nippon Gene, Tokyo) according to the manufacture's instructions. The RNA (1.0 μg) was subjected to RT-PCR using the primers for TS and TK cDNA for 34 cycles (each cycle consisted of denaturing at 94°C for 40 seconds, annealing at 55°C for 40 seconds and extension at 72°C for 40 seconds) in a Gene Amp PCR System 2400(Perkin Elmer, Branchburg, NJ, USA). RT-PCR was carried out with three sets of primers(TS: 5'-TGAATGGGGAGCTATCTTGCCA-3' and 5'-TCGTTGGATGTGG-ATTATACCC-3'; TK: 5'-TAGCACAGGCGGCACACGGAGT-3' and 5'-TGCTCCGCG-ATGTGACCCAGGA-3'; and β-actin: 5'-AGGCCCAGAGCAAGAGAGGCAT-3' and 5'-CATGGCTGGGGTGTTGAAGGTC-3'). The levels of TS mRNA and TK mRNA were determined by densitometry from photographs taken with an image analyzer (AE6920-MF Densitograph, ATTO, Tokyo), and are expressed as a ratio of the mRNA level of β-actin as an internal standard.

### Statistical analysis

All parameters were expressed as the mean ± standard error (SEM). Statistical analyses were performed using the unpaired *t*-test and one-way analysis of variance (ANOVA). The p values less than 0.05 were considered to be statistically significant.

## Results

### Body growth

There were no differences in mean body weight between the two groups for 2 weeks after the start of the study, i.e. at age 4 and 5 weeks. However, 3 weeks into the study, i.e. at the age of 6 weeks, mean body weight in the low-zinc diet group was less than 90% of the control value (p < 0.01), and as the experiment continued, it dropped to approximately 80% (p < 0.01) (data not shown).

### Organ weights

Although there was little difference in the wet weight of the adrenals, the wet weights of the spleen, liver, kidney, testis, ventral prostate and femur were markedly lower in the low-zinc diet group than the control group (p < 0.01) (data not shown).

### Hemograms

Concentrations of hemoglobin (Hb) and hematocrit (Ht), and numbers of erythrocytes (RBC), leukocytes (WBC) and platelets (Pt) were lower in the low-zinc diet group than the control group (p < 0.01 and 0.05) (data not shown).

### Plasma levels of biochemical markers and hormones

Although plasma levels of glucose, free fatty acid and triglyceride were not affected by the low-zinc diet, plasma level of total protein, albumin, total cholesterol and phospholipid were lowered compared with the control (p < 0.01 and 0.05) (Table 1). The activities of glutamic-oxaloacetic transaminase and lactate dehydrogenase were altered by the low-zinc diet though the differences were not statistically significant. Alkaline phosphatase activity in animals fed the low-zinc diet was markedly reduced to less than 60% of that in the controls (p < 0.01). Although there were no differences in plasma concentrations of sodium (Na), chloride (Cl), potassium (K), calcium (Ca) and inorganic phosphorus (iP) between the groups, the zinc (Zn) concentration in rats fed the low-zinc diet was markedly reduced to less than 60% of that in the controls (p < 0.01). There were no differences in plasma levels of growth hormone and corticosterone between the groups. However, plasma levels of luteinizing hormone and testosterone were lowered by the low-zinc diet (p < 0.01).

**Table 1:** Plasma biochemical markers and hormones

Groups (n)	**Control diet **(10)	**Low-zinc diet **(10)
Total protein (g/dL)	5.55 ± 0.23	4.39 ± 0.14**
Albumin (g/dL)	3.98 ± 0.20	3.23 ± 0.10**
Glucose (mg/dL)	166 ± 13	169 ± 17
		
Total cholesterol (mg/dL)	65.5 ± 1.8	53.3 ± 4.8*
Phospholipid (mg/dL)	136 ± 5	106 ± 7**
Free fatty acid (mEQ/L)	735 ± 163	409 ± 31
Triglyceride (mg/dL)	57.3 ± 25.3	28.5 ± 4.7
		
Blood urea nitrogen (mg/dL)	11.9 ± 0.8	12.7 ± 0.7
Creatinine (mg/dL)	0.17 ± 0.01	0.17 ± 0.02
		
AST (GOT) (IU/L)	147 ± 19	204 ± 30
Lactate dehydrogenase (IU/L)	571 ± 188	263 ± 63
Alkaline phosphatase (IU/L)	883 ± 40	497 ± 62**
		
Sodium (Na) (mEQ/L)	139 ± 2	142 ± 1
Chlorine (Cl) (mEQ/L)	99.6 ± 1.9	100.5 ± 1.9
Potassium (K) (mEQ/L)	4.74 ± 0.30	3.97 ± 0.20
Calcium (Ca) (mg/dL)	9.51 ± 0.26	9.12 ± 0.30
inorg. Phosphorus (iP) (mg/dL)	4.50 ± 0.36	3.82 ± 0.25
Zinc (Zn) (mg/dL)	107.2 ± 0.8	61.8 ± 4.2**
		
Growth hormone (ng/mL)	7.66 ± 1.96	6.57 ± 0.90
Luteinizing hormone (ng/mL)	3.07 ± 0.55	1.37 ± 0.14**
Testosterone (ng/mL)	1.26 ± 0.16	0.31 ± 0.07**
Corticosterone (ng/mL)	427 ± 48	418 ± 43

Data are means ± SEM.

** and *Significantly different from that of the Control; p < 0.01 and 0.05, respectively.

### In vivo BrdU labeling in prostate

**Figure 1:**
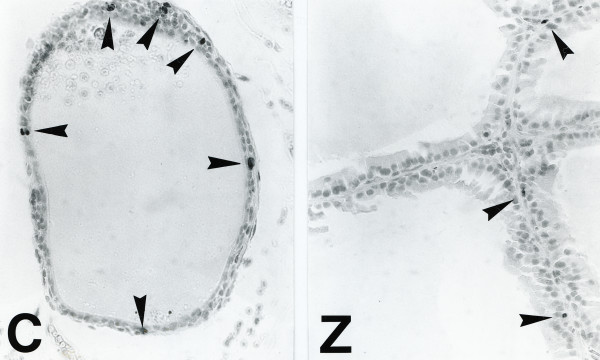
**In vivo BrdU labeling in the prostate of rats fed a normal control (C: 2.25 ± 0.40%) and a low-zinc (Z: 0.68 ± 0.19%) diets.** (Original magnification × 200).

Although BrdU-immunoreactive cells amounted to 3.19 ± 0.69% of the total cells in the prostate of the normal control (Fig. 1C), that percentage decreased to only 1.31 ± 0.48% in the prostate of rats fed the low-zinc diet (p < 0.05) (Fig. 1Z).

### Expression levels of TS and TK mRNA in prostate

**Figure 2:**
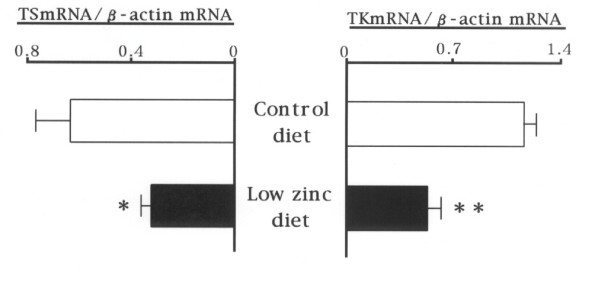
Expression levels of thymidylate synthase (TS: left sided) and thymidine kinase (TK: right sided) mRNA in the prostate of rats fed a normal control (upper bar) and a low-zinc (lower bar) diets.

The mRNA expression levels of prostatic thymidylate synthase (p < 0.05) and thymidine kinase (p < 0.01) were markedly reduced to 50% of the normal control by the low-zinc diet (Fig. 2).

## Discussion

Zinc is required for many biological functions including DNA synthesis, cell division, gene expression and the activity of various enzymes in all animals. A deficiency of zinc due to nutritional factors and several disease states is now recognized as a disorder. The high phytate content of cereal proteins is known to decrease the availability of zinc, thus the prevalence of zinc-deficiency is likely to be high in a population consuming large quantities of cereal proteins [5]. Zinc-deficient Bangladeshi infants showed improvements in rate of growth and a reduced incidence of acute lower respiratory infections after zinc supplementation, which produced highly significant, positive responses in height and weight [6]. Zinc-deficiency has had negative effects on growth rate, food intake, specific organ weights, hematological parameters, and serum levels of zinc, copper and iron, especially in rats fed the lowest zinc level [7].

In the present study, we investigated the effects of a low-zinc diet on body growth, organ weights, hemograms, plasma biochemical markers, plasma levels of hormones, and the BrdU-incorporation and TS and TK mRNA expression into the prostate cells in growing rats. The low-zinc diet caused a marked reduction in the body growth rate and weights of the spleen, liver, kidney, testis, ventral prostate and femur, along with a marked decline in the plasma concentration of zinc (less than 60% of the control), compared with the control diet. It resulted in a low hematopoiesis, hypoalbuminemia and hypocholesterolemia, thought it did not affect the plasma concentration of glucose. Although there were few differences in plasma biochemical markers, plasma levels of luteinizing hormone and testosterone were markedly reduced by six-week's feeding of a low-zinc diet. In prostate, BrdU-immunoreactive (S-phase) cells, and TS and TK mRNA expression levels were markedly affected by a zinc deficiency. TK activity increases during G_1 _and early S phases of the cell cycle and is often used as a marker of cell proliferation. TK is not a zinc metalloenzyme, but the transcription of the enzyme appears to be regulated by zinc availability. Zinc appears to regulate TK mRNA through zinc-dependent protein binding to the promoter region of the gene [8]. The decrease in TK activity occurred before a decline in food intake or body weight, and therefore was not associated with decreased nutrient availability.

In conclusion, a low-zinc diet appears to reduce the body growth and organ weights including prostate, causing low plasma levels of luteinizing hormone and testosterone and reduction in prostate DNA replication in growing-rats.

## Competing interests

The authors declare that they have no competing interests.

## Authors' contributions

YI has made substantial contributions to conception and analysis of data, and carried out the animal experiments under the guidance of SAS, and has involved in drafting the manuscript or revising it. HK participated in drafting the manuscript. SUZ carried out the immunochemistry using BrdU in rat prostate. NN performed the statistical analysis. SAS performed the determination of expression levels of prostatic TS and TK mRNA. SAK conceived of the study, and participated in its design and coordination and helped to draft the manuscript. All authors read and approved the final manuscript.
